# Testing the Water–Energy Theory on American Palms (Arecaceae) Using Geographically Weighted Regression

**DOI:** 10.1371/journal.pone.0027027

**Published:** 2011-11-03

**Authors:** Wolf L. Eiserhardt, Stine Bjorholm, Jens-Christian Svenning, Thiago F. Rangel, Henrik Balslev

**Affiliations:** 1 Ecoinformatics and Biodiversity Group, Department of Bioscience, Aarhus University, Aarhus, Denmark; 2 Departamento de Ecologia, ICB, Universidade Federal de Goiás, Goiânia, GO, Brazil; Michigan State University, United States of America

## Abstract

Water and energy have emerged as the best contemporary environmental correlates of broad-scale species richness patterns. A corollary hypothesis of water–energy dynamics theory is that the influence of water decreases and the influence of energy increases with absolute latitude. We report the first use of geographically weighted regression for testing this hypothesis on a continuous species richness gradient that is entirely located within the tropics and subtropics. The dataset was divided into northern and southern hemispheric portions to test whether predictor shifts are more pronounced in the less oceanic northern hemisphere. American palms (Arecaceae, *n* = 547 spp.), whose species richness and distributions are known to respond strongly to water and energy, were used as a model group. The ability of water and energy to explain palm species richness was quantified locally at different spatial scales and regressed on latitude. Clear latitudinal trends in agreement with water–energy dynamics theory were found, but the results did not differ qualitatively between hemispheres. Strong inherent spatial autocorrelation in local modeling results and collinearity of water and energy variables were identified as important methodological challenges. We overcame these problems by using simultaneous autoregressive models and variation partitioning. Our results show that the ability of water and energy to explain species richness changes not only across large climatic gradients spanning tropical to temperate or arctic zones but also within megathermal climates, at least for strictly tropical taxa such as palms. This finding suggests that the predictor shifts are related to gradual latitudinal changes in ambient energy (related to solar flux input) rather than to abrupt transitions at specific latitudes, such as the occurrence of frost.

## Introduction

Among the contemporary environmental factors that are correlated with species richness on broad scales, water and energy have emerged as key influences [1]–[4]. However, these two factors do not appear to be equally important worldwide. Based on a review of 85 studies of broad-scale richness gradients, Hawkins et al. [2] hypothesized that ‘the relative importance of the two components of water–energy dynamics shifts latitudinally’ (p. 3111). This conjecture has not received much attention (but see [5]), although appropriate tools exist for exploring spatial non-stationarity in environment–richness relationships [6]–[10].

The question of how patterns of species richness are controlled has been debated for decades [11], [12] and remains a central issue in macroecological and biogeographical research [13]–[15]. Various explanations have been proposed that emphasize the importance of area [16], geometric constraints [13], history [17], synergism between climate and history [18], and, most commonly, contemporary environment [2], [3], [19]. Many studies have focused on the role of contemporary climate as the main predictor of species richness, concluding in favor of a central role for water- and energy-related variables [2], [4], [19], [20]. Different mechanisms have been proposed to explain the suggested primacy of water and energy, including trophic, physiological, and metabolic effects [2], [21], [22]. Fundamentally, a dynamic relationship between energy and water may result from life's dependence on both liquid water and ambient energy [2], [4], [20].

Of interest, richness gradients at low latitudes appear to correlate most strongly with water availability, while energy (for animals) or water–energy variables (for plants) are the best correlates of most richness gradients at high latitudes [2]. This pattern finds a convincing *a posteriori* explanation in the latitudinal gradient of solar flux. Accordingly, energy is expected to be the most limiting factor at high latitudes where energy levels are low, while water gradients should be more important at low latitudes where ambient energy is high and thus not limiting [2]. For plants, the mechanism causing this predictor shift is thought to be related to physiological processes, while for animals it is more difficult to determine whether the shift results from direct physiological effects or from plant productivity [2], [4], [23]. Worth noting, the pattern appears to be asymmetric, with predictor shifts being largely restricted to the northern hemisphere, whereas water is more important than energy in most southern hemisphere regions [2]. A potential explanation may lie in the more oceanic climates of the southern hemisphere [2]. Increasing evidence also suggests that climate–richness relationships depend on evolutionary processes (e.g., [24]), which may introduce spatial non-stationarity. In particular, phylogenetic niche conservatism can cause groups to be most species rich in their ancestral climates [25]. To the extent that different groups originate from different climates, this mechanism may lead to climate–richness relationships that vary among groups and regions [24].

Since the meta-analysis by Hawkins et al. [2], only one study has formally tested the hypothesized predictor shifts and found support for it for plants and animals in Europe [5]. The questions of whether predictor shifts are abrupt or gradual, at which latitudes they occur, whether they can be related to specific climatic transitions (such as the subtropical-temperate boundary), and to what degree those parameters are taxon-specific await further investigation.

Geographical shifts in the explanatory power of environmental predictors are broadly relevant to macroecological research because they pose a challenge to ‘global’ models of biodiversity. Regression techniques that are typically used to analyze environment–richness relationships, such as ordinary least squares regression [1], generalized additive models (e.g. [5]), or spatial autoregressive models [26], assume that the relationship is described by one set of parameters that applies equally throughout the study area. It has been argued that such models are misleading if the analyzed relationship is indeed variable in space (spatial non-stationarity) [6] (but see [27]). If the purpose is simply to identify correlates of richness, the problem can be referred to the scale-dependency of environment–richness relationships [28], with different correlations on global and smaller scales correctly describing a given pattern. If, however, the purpose is to understand the actual drivers of richness, the ‘average’ parameters obtained from a global model [6], [29] might not be informative if the driving dynamics occur at a scale smaller than the model. It is therefore relevant to explore both scale-dependency and spatial non-stationarity of such relationships [6], [7]. Specifically, spatial non-stationarity is a promising source of information because the relationship as such can be related to (second-order) predictor variables.

Geographically weighted regression (GWR) is a geographically local modeling technique specifically designed to deal with spatial non-stationarity in the modeled relationships [8]. GWR performs one weighted ordinary least squares regression per observation in the analyzed dataset. Weights are applied as a (typically inverse) function of the distance from the location of the ‘focal’ data point. Of importance, modeling is carried out at a scale smaller than the study extent, defined by the distance decay (‘bandwidth’) of the weighting function. Thus, GWR should not be used as an alternative to global regression models but as a complementary technique for quantifying spatial variability (non-stationarity) in relationships between the predictor and response variables [27]. By allowing regression model parameters to vary in space and then mapping these coefficients, GWR makes it possible to quantify and test the spatial variability in the species–environmental relationships. GWR is increasingly used for analyzing species richness patterns [6], [10], [22], [29], [30], [31], and some of these studies [22], [29], [30] have produced results that are in agreement with the predictor shifts conjectured by Hawkins et al. [2]. However, no study has to date applied GWR in a formal test of this hypothesis.

Using GWR, we tested for predictor shifts not by comparing disparate studies from different regions, but by quantifying geographic variation in the ability of water and energy to explain the species richness of a single group of organisms (palms) across a continuous region, the American tropics and subtropics. Palms are a diverse, pan-tropical family of ca. 2,400 species worldwide [32]. They are important constituents of many vegetation types in tropical, subtropical, and, more rarely, warm-temperate parts of the New World [33]. Several previous studies have investigated the controls of the large-scale diversity patterns in American palms and have found water-related variables to be of primary importance among contemporary environmental factors [34], [35]. Thus, as a low-latitude group when regarded on a global scale, the palms conform well to the conjecture of Hawkins et al. [2]. However, here we focus on assessing whether the relative importance of water and energy also changes with latitude within the range of palms. Moving away from the tropics, the American palms are likely to become more controlled by available energy than many other plant groups because of key aspects of palm architecture and anatomy that have been previously described [32], [36], [37]. Thus, they are good candidates for displaying latitudinal predictor shifts sensu Hawkins et al. [2] at relatively low latitudes. Here, we used GWR to formally test the following two predictions for a species-rich organism group within the tropics/subtropics: (1) Temperature is a stronger correlate of palm species richness at high latitudes than at low latitudes, while water shows the opposite trend, as hypothesized by Hawkins et al. [2]; (2) this latitudinal shift is strongest in the northern hemisphere, reflecting the more oceanic southern hemispheric climates.

## Methods

### Study species and area

We used distribution data for the complete palm family (Arecaceae) across the Americas (*n* = 547 spp.) extracted using ArcView 9.2 (ESRI Inc., Redlands, California, USA) from the range maps in the *Field Guide to the Palms of the Americas* [38]. The number of palm species present was registered for all cells of a continuous grid covering the whole range of palms in the Americas ranging from 34° North to 33° South. Based on the quality of the maps and our knowledge of the distribution of the palm family, we decided to work at a resolution of 1°×1° grid cells. Cells with less than 25% land surface were excluded from the analysis because their species richness might be more strongly determined by area than by climate. Moreover, we excluded 59 also mostly coastal grid cells for which climate variables (see below) were not available. These criteria resulted in 1510 grid cells across the Americas (Figure S1).

### Environmental variables

As predictors, we used the environmental variables of mean annual precipitation (AP), minimum precipitation of the driest month (MPDM), mean annual temperature (MAT), and minimum temperature of the coldest month (MTCM) from the WorldClim global climate database [39] at a resolution of 30 arc seconds (http://www.worldclim.org/current). Moreover, we used potential evapotranspiration (PET) and actual evapotranspiration (AET) from the 30 arc minutes resolution UNEP GNV183 data set (www.grid.unep.ch/GRID_search_details.php?dataid=GNV183/) [40]. To match the resolution of the palm grid cells, the average of each variable was taken for the terrestrial part of each 1°×1° grid cell using ArcInfo 10 (ESRI Inc., Redlands, California, USA). AET was only used to calculate water deficit (WD  =  PET – AET), representing drought [10]. We did not use AET directly because it represents both water and energy [41], running counter to the study purpose of separating these two aspects of climate. Thus, we worked with two sets of variables, one set denoting water (AP, MPDM, and WD), and one denoting energy (MAT, MTCM, and PET). Non-climatic variables were not included because the explicit aim was to infer the roles of water and energy, not to explain as much variation in species richness as possible (cf. [5]).

### Statistical analyses

The software SAM [42] was used to fit GWR models using palm species richness as the response variable and different combinations of climatic variables as predictors. In a first step, we performed information-theoretic model selection using the corrected Akaike information criterion (AIC_C_; [43]) to determine which combination of predictor variables had the highest explanatory power within each set of climatic variables (water and energy, respectively). In each set, AIC_C_ was calculated for all possible combinations of one to three predictor variables.

We then computed local R^2^ values for the best water model, the best energy model, and a model including the predictor variables of both the best water model and the best energy model (‘combined model’ in the following). These values represent the fraction of local variation in palm richness explained by water (R_w_) and energy (R_e_). For each grid cell, variation partitioning [44], [45] was performed to determine the amount of variation that is uniquely explained by water and energy. Those fractions were calculated as R_pe_  =  R_t_ – R_w_ for pure energy and R_pw_  =  R_t_ – R_e_ for pure water, where R_t_ is the local R^2^ value of the combined model.

The results of GWR depend on the choice of the spatial kernel function that determines how observations are weighted as a function of spatial distance from the focal cell [6], [8], [46], [47]. To ensure that our conclusions did not depend on a specific choice of this function, we repeated all GWR analyses with four different kernels. First, we used the bi-square function, which applies a continuous, near-Gaussian weighting function up to a distance *b* (the ‘bandwidth’) from the regression point and then zero weights to any observation beyond *b*. Two values of *b* were used, 1200 km and 1800 km. Second, we used the moving window approach, which assigns equal weights to observations within the bandwidth and zero to observations beyond [8], with the same two *b* values. The bandwidths were chosen based on the resolution of the palm diversity data (1° × 1°, i.e., 111 km × 110 km at the equator) and the total extent of the study area (approx. 10,000 km between the most northerly and most southern data points) to ensure a reasonable local sample size and a scale that was clearly local relative to the whole study area. Because we were specifically interested in responses from the marginal areas (alone and not lumped together to obtain a certain number of grid cells), we did not use adaptive spatial kernels, which adapt the bandwidth according to the variation in observations so that it is large in areas with low density of data and smaller in areas with plenty of observations [8].

**Figure 1:**
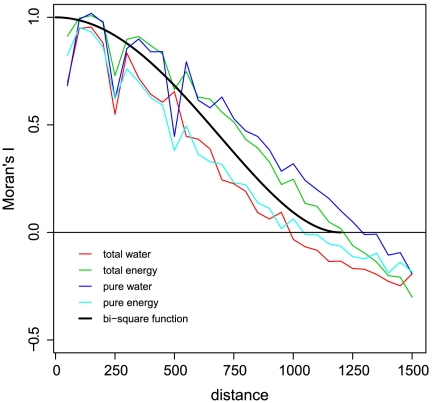
Inherent spatial autocorrelation of local GWR results. Moran's I correlogram of the amount of variation in American palm species richness locally explained by water and energy in geographically weighted regression. The black line shows the kernel function of the GWR analysis for comparison, a bi-square function with a bandwidth of 1200 km. Distance in km.

To determine how the amount of local variation in palm richness explained by water and energy changes with latitude, we regressed R_e_, R_pe_, R_w_, and R_pw_ on absolute latitude separately for the northern and southern hemispheres. This was done using both ordinary least squares (OLS) regression and simultaneous autoregressive (SAR) models [26]. The local results of GWR are inherently spatially autocorrelated because the local results for geographically close cells are based on overlapping datasets. Spatial autocorrelation may cause false significance of parameter estimates or bias the parameter estimates themselves, and it has even been reported to sometimes invert the relationship between predictor and response when standard OLS regression is used [26], [48]. Here, the spatial scale of inherent autocorrelation was related to the GWR kernel function (Fig. 1). The neighborhood and distance weighting for the SAR models was thus implemented using the same function as for the GWR kernel; for the local results of the 1200-km bi-square GWR, for example, we used a neighborhood of 1200 km and weighted the cells according to the bi-square function. Lagged SAR models were used because these are designed to model inherent (as opposed to induced) spatial autocorrelation [26]. All regression analyses were carried out in R 2.10.1 [49]; the package spdep 0.5-31 (http://cran.r-project.org/web/packages/spdep/index.html) was used for the SAR analyses.

## Results

**Figure 2:**
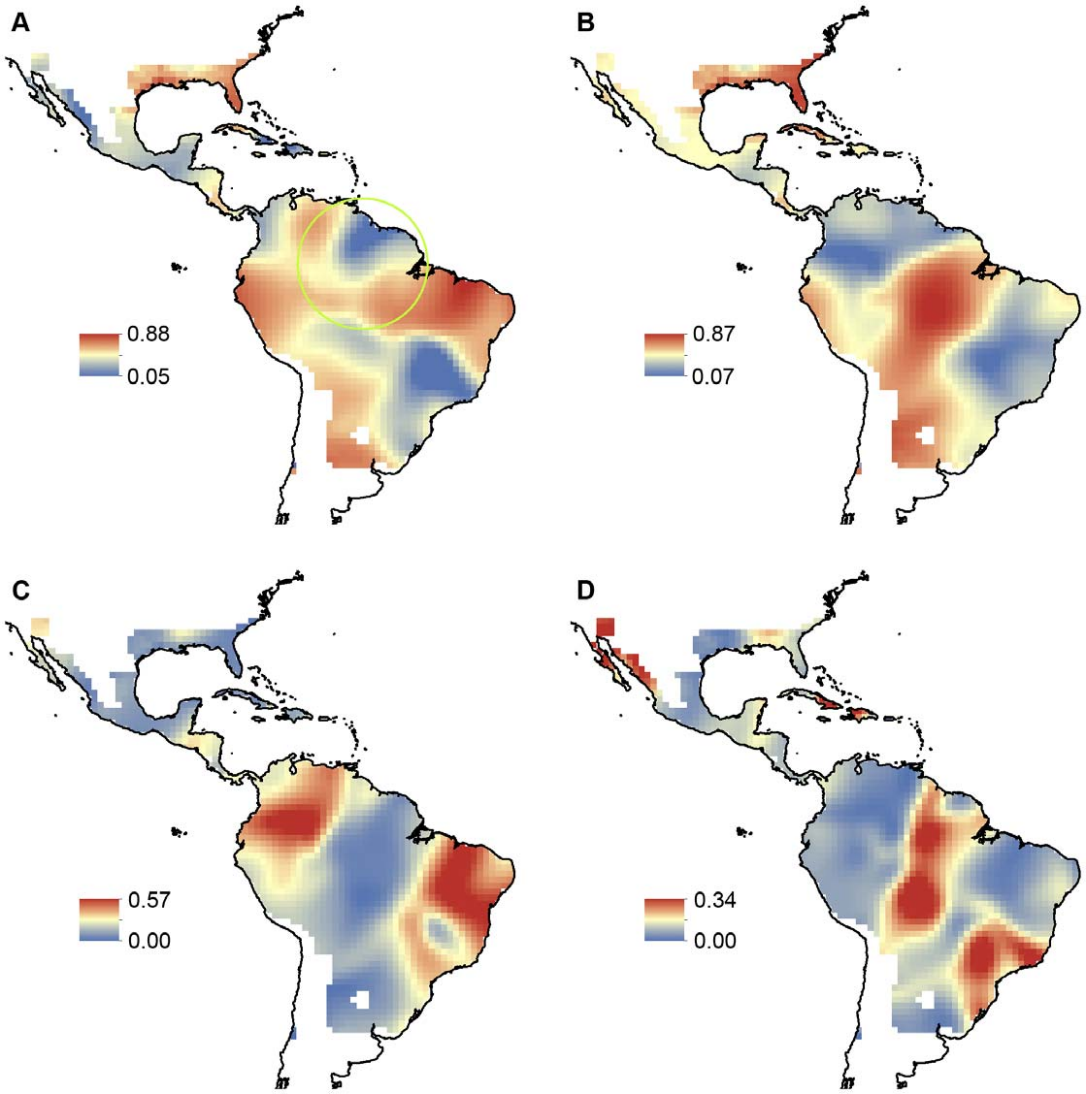
Variation in American palm species richness locally explained by water and energy. Local R^2^ values obtained from geographically weighted regression (GWR) of palm species richness on annual precipitation, precipitation of the driest month, and water deficit (A) and mean annual temperature, minimum temperature of the coldest month, and potential evapotranspiration (B). Fraction of variation uniquely explained by the water variables (C) and energy variables (D) obtained from variation partitioning. The green circle in (A) shows the GWR bandwidth for a cell situated at the equator.

**Figure 3:**
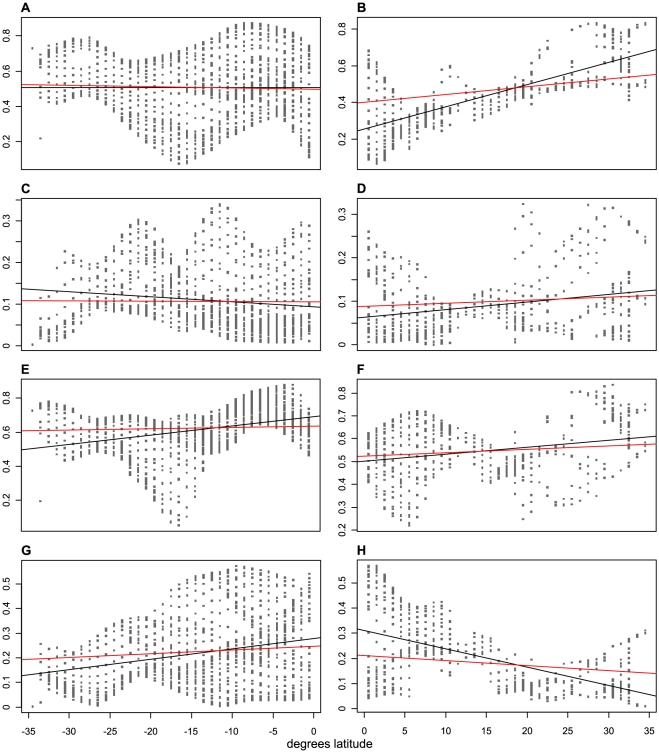
Latitudinal trends in the ability of water and energy to explain American palm species richness. The amount of variation in palm species richness locally explained by energy variables (A–D) and water variables (E–G) plotted against latitude. A, B: total energy (R_e_). C, D: pure energy (R_pe_). E, F: total water (R_w_). G, H: pure water (R_pw_). Regression lines obtained from OLS regression (black) and SAR regression (red).

There was strong spatial heterogeneity in palm richness–climate relationships, as evidenced by a minimum AIC_C_ difference between GWR and OLS models of ΔAIC_C_  =  663 (median 1515, maximum 3043) (Tables S1, S2, S3, S4). The model selection procedure clearly favored the models containing all water variables and all energy variables, respectively, with AIC_C_ differences of 108–179 and 103–146, depending on the GWR kernel, to the next best model (Tables S1, S2, S3, S4). Local R^2^ values also provided evidence for strong spatial heterogeneity in the importance of water and energy (Fig. 2). Of note, evidence was consistent for a decrease in the unique explanatory power of water (R_pw_) with absolute latitude and a simultaneous increase in the unique explanatory power of energy (R_pe_; Tables 1 and 2, Fig. 3). Latitudinal trends in the amount of variation of palm richness that is locally explained by water or energy (Fig. 3) were largely robust to the choice of models, i.e., the spatial kernel used in GWR and the use of OLS vs. SAR models for evaluating the GWR results against latitude (Tables 1 and 2). Different model combinations produced no significant latitudinal trends of opposite sign, but relationships were non-significant in some cases (Tables 1 and 2).

**Table 1:** Latitudinal trends in the amount of variation in American palm species richness locally explained by water and energy (OLS).

		Bi-square		Moving window	
Model	Hemisphere	1200 km	1800 km	1200 km	1800 km
Energy (total)	North	**0.728**	**0.753**	**0.703**	**0.757**
Energy (pure)	North	**0.26**	**0.179**	**0.25**	**0.207**
Water (total)	North	**0.256**	**0.503**	**0.345**	**0.462**
Water (pure)	North	**−0.559**	**−0.665**	**−0.59**	**−0.827**
Energy (total)	South	**−**0.001	**0.285**	**0.11**	**0.259**
Energy (pure)	South	**0.14**	**0.185**	**0.235**	**0.482**
Water (total)	South	**−0.34**	**−0.157**	**−0.39**	**−0.448**
Water (pure)	South	**−0.246**	**−0.421**	**−0.369**	**−0.435**

Values are slopes of ordinary least squares regressions on standardized variables. Positive signs indicate increase with absolute latitude. Bold: p<0.05.

**Table 2:** Latitudinal trends in the amount of variation in American palm species richness locally explained by water and energy (SAR).

		Bi-square		Moving window	
Model	Hemisphere	1200 km	1800 km	1200 km	1800 km
Energy (total)	North	**0.256**	**0.245**	0.017	**0.059**
Energy (pure)	North	**0.104**	**0.085**	**0.078**	0.069
Water (total)	North	**0.128**	**0.18**	**−**0.022	**−**0.041
Water (pure)	North	**−0.151**	**−0.189**	**−**0.007	**−0.129**
Energy (total)	South	**0.037**	**0.144**	0.027	**0.115**
Energy (pure)	South	0.013	**0.07**	0.041	**0.204**
Water (total)	South	**−0.051**	**−0.049**	**−0.079**	**−0.191**
Water (pure)	South	**−0.088**	**−0.195**	**−0.083**	**−0.186**

Values are slopes of simultaneous autoregressive models using standardized variables. Positive signs indicate increase with absolute latitude. Bold: p<0.05.

As was apparent from the mapping of the local R^2^ values and fractions of variation uniquely explained by water and energy (Fig. 2), the GWR results exhibited strong spatial autocorrelation (Fig. 1), as expected based on the functional principles of GWR. However, comparison of the OLS and SAR results showed that this autocorrelation did not qualitatively affect the estimated latitudinal relationships (Tables 1 and 2, Fig. 3).

## Discussion

Water–energy dynamics theory predicts that species richness is primarily controlled by the availability of water and ambient energy, with water being most influential at low latitudes and energy being most influential at high latitudes [2]. Results from our tests using American palms as a model group strongly support this latitudinal predictor shift (Tables 1 and 2). Similar water–energy predictor shifts have also been found for European mammals, birds, amphibians, and plants [5]. Whittaker et al. [5] used global modeling techniques on separate northern and southern datasets and found that energy had a relatively larger contribution to explained variance in northern data. Evidence also indicates that the relationship between Australian pteridophyte richness and water becomes weaker towards higher latitudes, while the relationship with temperature becomes stronger [30]. Similar conclusions have been drawn for American amphibians [29] and European dragonflies [22]. These three studies used GWR but did not involve statistical analysis of the resulting spatial patterns.

Here, we moved beyond previous tests of water–energy dynamics theory in several ways. Water–energy predictor shifts were originally observed between tropical/subtropical areas, where water is the dominant environmental predictor of plant species richness, and temperate areas, where energy or water–energy variables are most influential [2]. For detection of such a shift, the analysis must include a part of the climatic gradient in which energy is clearly limiting for the studied taxon (either in terms of physiology, productivity, or food availability). In the previously studied taxa, this shift seems to occur at relatively high latitudes, usually north of the transition between subtropics and tropics (see [2], [5] for examples). Our results show that latitudinal predictor shifts can also occur within the tropical/subtropical zone, at least for megathermal taxa such as palms. Palms are thought to be maladapted to meso- or microthermal climates because of their soft and water-rich tissue, their inability to undergo dormancy, and their lack of physiological adaptations to frost [37]. These characteristics are obviously highly phylogenetically conserved, making the palms a prime example of a group that fits the tropical conservatism hypothesis [25]. Palms exhibit a strong latitudinal gradient of diversity in the Americas, with highest diversity close to the equator and no species beyond 34°Ν and 34°S [34], [35], [50]. In line with the expectations of water–energy dynamics theory for low latitudes, the broad-scale pattern of palm diversity in the Americas is best explained by water [34], [35], but energy plays an additional role [50]–[52]. Given that the latitudinal limits of palm occurrence are almost certainly set by low temperatures [53], an influence of energy especially on the high-latitude tails of the diversity gradient is plausible.

The prediction that the latitudinal predictor shift is strongest in the northern hemisphere [2] is not supported by our results. Neither the magnitude nor the significance of the latitudinal trends in variable importance differ systematically between the hemispheres (Tables 1 and 2). This finding is likely due to our study being restricted to tropical and subtropical latitudes, where climatic gradients apparently do not differ strongly enough between the hemispheres to entail significant differences in latitudinal predictor shifts. Such differences may still be found in taxa that extend into temperate or arctic zones.

Previous groups have tested for latitudinal differences in the predictive power of variables by dividing datasets into latitudinal bands [2], [5]. Central to this approach is finding the appropriate latitudinal threshold at which to split the dataset, and interpretation relies on the assumption that relationships are stationary within each latitudinal band. If this is not the case, ‘global’ models such as OLS or SAR models provide average estimates that can be difficult to interpret because they may not apply to any of the location within the study region [6], [29]. GWR is specifically designed to deal with geographic non-stationarity of model coefficients [6]–[8]. It is therefore a suitable approach to studying predictor shifts if no good argument can be made for dividing the dataset at a particular point, or spatial stationarity within the partial dataset is not guaranteed.

A downside of GWR compared to the latitudinal-bands approach is that the datasets used for local models overlap excessively, resulting in strong spatial autocorrelation of local coefficient estimates (Fig. 1). Our results illustrate how difficult it is to visually interpret GWR results because of the high degree of inherent spatial autocorrelation; latitudinal trends are not that obvious when R^2^ values are mapped (Fig. 2). Moreover, OLS regressions that use local GWR results as the dependent variable are prone to bias because of the inherently strong spatial autocorrelation. The use of lagged SAR models is a way to overcome this bias [26], so that GWR results can be used to quantify the strength and shape of predictor shifts along latitudinal (or other) gradients.

GWR is also an efficient tool for exploring the scale-dependency of relationships [6], [7], [27]. Predictors of species richness are thought to vary systematically with spatial scale [28]; this effect is also well documented for palms [36]. Predictor shifts sensu Hawkins et al. [2] might therefore depend on the scale at which climate–richness relationships are quantified. In the present study, the GWR bandwidth defined this scale. Latitudinal trends in energy and water effects on palm richness emerged irrespective of the scale of the GWR analysis (1200 km vs. 1800 km bandwidth). Smaller bandwidths were not used because GWR tends to over-fit at very small scales, leading to unrealistic R^2^ values [27]; and larger bandwidths were not used because they would approach the extent of the total dataset and therefore not allow for sufficient geographic variability. However, our results indicate that the observed latitudinal trends are not restricted to a certain spatial scale.

To our knowledge, no previous study, whether it used global or local modeling techniques, has quantified the independent effects of water and energy on species richness. Our results show that taking into account parallel or synergistic effects of water and energy can strongly influence conclusions when testing for predictor shifts. When the water was analyzed irrespective of energy (“total water,” Tables 1 and 2), the influence of water increased with latitude in the northern hemisphere in contrast to the predictions [2]. However, this finding for the northern hemisphere seems to be the result of an interaction with energy. When variation partitioning [44], [45] was used to identify the amount of local variation in palm species richness that is uniquely explained by water-related variables (“pure water,” Tables 1 and 2), the expected negative relationship emerged also for the northern hemisphere. This finding suggests that studies that compare the explanatory power of variables (or sets of variables) without taking the interactions of these variables into account (e.g., [30], [54]) must be interpreted with caution.

Scale dependency and spatial non-stationarity are prevalent features of environment–richness relationships and require consideration in the effort to explain spatial patterns of species diversity. Parallel to the current progress in finding global determinants of diversity (e.g., [55]) and understanding their scaling (e.g., [56]), evidence is accumulating for predictable patterns of spatial non-stationarity. Increases in the predictive power of water and decreases in the predictive power of energy variables with absolute latitude have been documented across continents, climatic zones, and taxa [2], [5], [22], [29], [30]; the current work now extends that to the tropics/subtropics. However, more exploration is needed into the universality of this relationship, its shape, and its variation across taxa. GWR or similar local modeling techniques are more powerful tools for this task than traditional ‘global’ models but are not without issues, and further statistical development is desirable, especially concerning spatial autocorrelation both at the level of single (local) models and the overall GWR fit.

## Supporting Information

Figure S1 **Maps of American palm species richness and climatic variables.** (A) Palm species richness, (B) mean annual temperature, (C) mean temperature of the coldest month, (D) potential evapotranspiration, (E) actual evapotranspiration, (F) water deficit, (G) annual precipitation, and (H) minimum precipitation of the driest month.
(TIF)

Table S1 Model selection for GWR with bi-square kernel, *b* = 1200 km. AP: annual precipitation; MPDM: minimum precipitation of the driest month; WD: water deficit; MAT: mean annual temperature; MTCM: minimum temperature of the coldest month; PET: potential evapotranspiration; ΔAIC_C_ is the difference between the corrected Akaike information criterion values of two models; GWR: geographically weighted regression; OLS: ordinary least squares regression; *Best water model/best energy model.
(DOC)

Table S2 Model selection for GWR with bi-square kernel, *b* = 1800 km. AP: annual precipitation; MPDM: minimum precipitation of the driest month; WD: water deficit; MAT: mean annual temperature; MTCM: minimum temperature of the coldest month; PET: potential evapotranspiration; ΔAIC_C_ is the difference between the corrected Akaike information criterion values of two models; GWR: geographically weighted regression; OLS: ordinary least squares regression; *Best water model/best energy model.
(DOC)

Table S3 Model selection for GWR with moving window kernel, *b* = 1200 km. AP: annual precipitation; MPDM: minimum precipitation of the driest month; WD: water deficit; MAT: mean annual temperature; MTCM: minimum temperature of the coldest month; PET: potential evapotranspiration; ΔAIC_C_ is the difference between the corrected Akaike information criterion values of two models; GWR: geographically weighted regression; OLS: ordinary least squares regression; *Best water model/best energy model.
(DOC)

Table S4 Model selection for GWR with moving window kernel, *b* = 1800 km. AP: annual precipitation; MPDM: minimum precipitation of the driest month; WD: water deficit; MAT: mean annual temperature; MTCM: minimum temperature of the coldest month; PET: potential evapotranspiration; ΔAIC_C_ is the difference between the corrected Akaike information criterion values of two models; GWR: geographically weighted regression; OLS: ordinary least squares regression; *Best water model/best energy model.
(DOC)
