# Supplementary figures and images for: Testing the Water–Energy Theory on American Palms (Arecaceae) Using Geographically Weighted Regression
Source: PLoS One. 2011 Nov 3;6(11):e27027. doi: 10.1371/journal.pone.0027027 (PMC3207816; doi:10.1371/journal.pone.0027027)

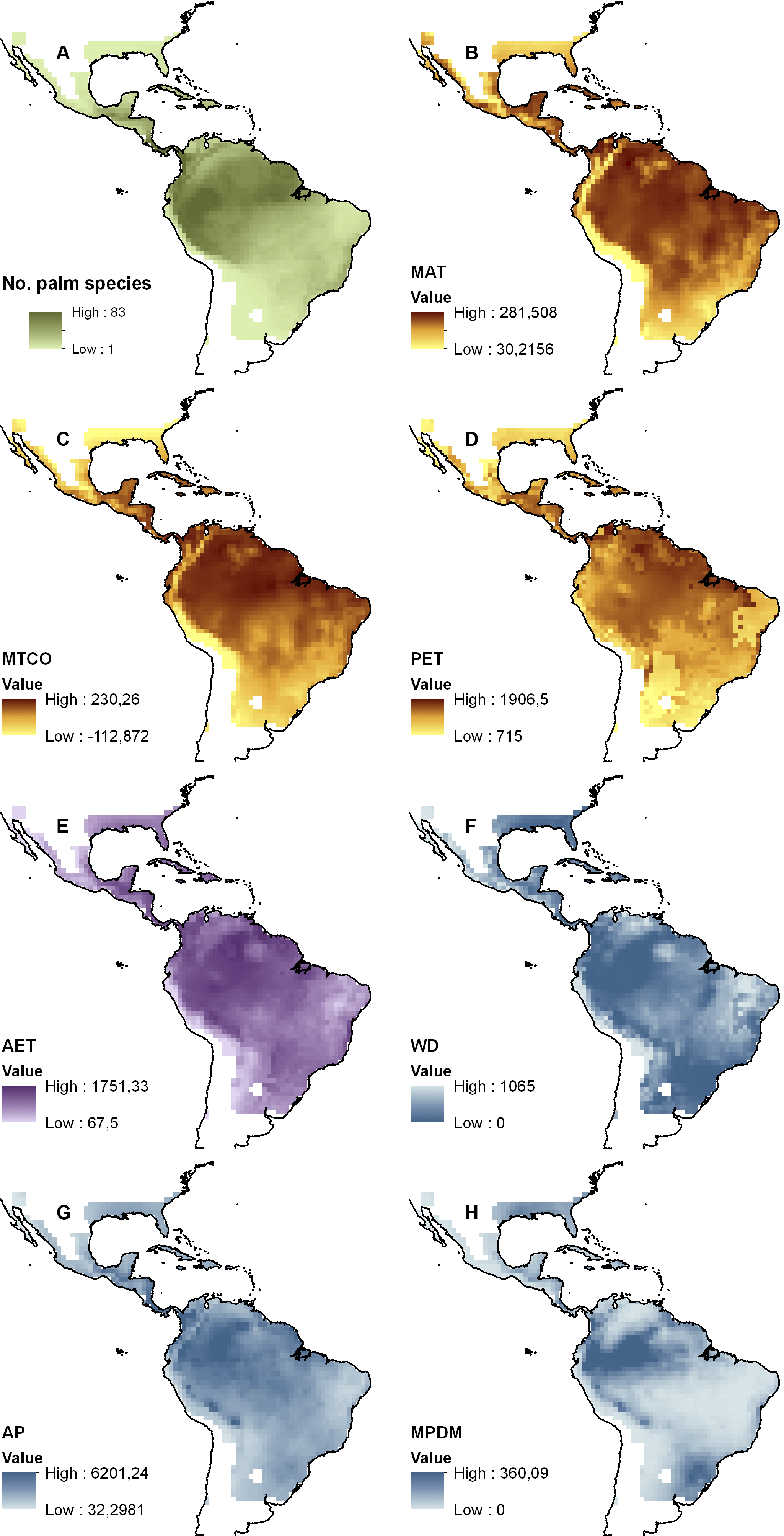

Supplement: Figure S1 — Maps of American palm species richness and climatic variables. (A) Palm species richness, (B) mean annual temperature, (C) mean temperature of the coldest month, (D) potential evapotranspiration, (E) actual evapotranspiration, (F) water deficit, (G) annual precipitation, and (H) minimum precipitation of the driest month. (TIF) [file pone.0027027.s001.tif]
