# Supplementary material for: Testing the Water–Energy Theory on American Palms (Arecaceae) Using Geographically Weighted Regression
Source: PLoS One. 2011 Nov 3;6(11):e27027. doi: 10.1371/journal.pone.0027027 (PMC3207816; doi:10.1371/journal.pone.0027027)
Supplement: Table S1 — Model selection for GWR with bi-square kernel, b = 1200 km. AP: annual precipitation; MPDM: minimum precipitation of the driest month; WD: water deficit; MAT: mean annual temperature; MTCM: minimum temperature of the coldest month; PET: potential evapotranspiration; ΔAICC is the difference between the corrected Akaike information criterion values of two models; GWR: geographically weighted regression; OLS: ordinary least squares regression; *Best water model/best energy model. (DOC) [file pone.0027027.s002.doc]

**Table S1. Model selection for GWR with bi-square kernel, *b* = 1200 km**.

| **Water** | | | **Energy** | | |  |  |
| --- | --- | --- | --- | --- | --- | --- | --- |
| **AP** | **MPDM** | **WD** | **MAT** | **MTCM** | **PET** | Δ**AIC_C_**  **to best GWR** | Δ**AIC_C_**  **to OLS** |
| × |  |  |  |  |  | 436 | 1746 |
|  | × |  |  |  |  | 374 | 1808 |
|  |  | × |  |  |  | 266 | 3043 |
| × | × |  |  |  |  | 220 | 1955 |
| × |  | × |  |  |  | 217 | 1951 |
|  | × | × |  |  |  | 76 | 2918 |
| × | × | × |  |  |  | 0* | 2146 |
|  |  |  | × |  |  | 783 | 2461 |
|  |  |  |  | × |  | 803 | 1959 |
|  |  |  |  |  | × | 710 | 1988 |
|  |  |  | × | × |  | 688 | 1642 |
|  |  |  | × |  | × | 597 | 2100 |
|  |  |  |  | × | × | 618 | 1943 |
|  |  |  | × | × | × | 493* | 1711 |

AP: annual precipitation; MPDM: minimum precipitation of the driest month; WD: water deficit; MAT: mean annual temperature; MTCM: minimum temperature of the coldest month; PET: potential evapotranspiration; ΔAIC_C_ is the difference between the corrected Akaike information criterion values of two models; GWR: geographically weighted regression; OLS: ordinary least squares regression; *Best water model/best energy model.
