# Supplementary material for: Testing the Water–Energy Theory on American Palms (Arecaceae) Using Geographically Weighted Regression
Source: PLoS One. 2011 Nov 3;6(11):e27027. doi: 10.1371/journal.pone.0027027 (PMC3207816; doi:10.1371/journal.pone.0027027)
Supplement: Table S2 — Model selection for GWR with bi-square kernel, b = 1800 km. AP: annual precipitation; MPDM: minimum precipitation of the driest month; WD: water deficit; MAT: mean annual temperature; MTCM: minimum temperature of the coldest month; PET: potential evapotranspiration; ΔAICC is the difference between the corrected Akaike information criterion values of two models; GWR: geographically weighted regression; OLS: ordinary least squares regression; *Best water model/best energy model. (DOC) [file pone.0027027.s003.doc]

**Table S2. Model selection for GWR with bi-square kernel, *b* = 1800 km**.

| **Water** | | | **Energy** | | |  |  |
| --- | --- | --- | --- | --- | --- | --- | --- |
| **AP** | **MPDM** | **WD** | **MAT** | **MTCM** | **PET** | Δ**AIC_C_**  **to best GWR** | Δ**AIC_C_**  **to OLS** |
| × |  |  |  |  |  | 541 | 1224 |
|  | × |  |  |  |  | 425 | 2156 |
|  |  | × |  |  |  | 352 | 2540 |
| × | × |  |  |  |  | 252 | 1506 |
| × |  | × |  |  |  | 320 | 1431 |
|  | × | × |  |  |  | 108 | 2469 |
| × | × | × |  |  |  | 0* | 1729 |
|  |  |  | × |  |  | 904 | 1923 |
|  |  |  |  | × |  | 876 | 1469 |
|  |  |  |  |  | × | 831 | 1450 |
|  |  |  | × | × |  | 722 | 1191 |
|  |  |  | × |  | × | 756 | 1525 |
|  |  |  |  | × | × | 752 | 1392 |
|  |  |  | × | × | × | 576* | 1211 |

AP: annual precipitation; MPDM: minimum precipitation of the driest month; WD: water deficit; MAT: mean annual temperature; MTCM: minimum temperature of the coldest month; PET: potential evapotranspiration; ΔAIC_C_ is the difference between the corrected Akaike information criterion values of two models; GWR: geographically weighted regression; OLS: ordinary least squares regression; *Best water model/best energy model.
