# Supplementary material for: Testing the Water–Energy Theory on American Palms (Arecaceae) Using Geographically Weighted Regression
Source: PLoS One. 2011 Nov 3;6(11):e27027. doi: 10.1371/journal.pone.0027027 (PMC3207816; doi:10.1371/journal.pone.0027027)
Supplement: Table S3 — Model selection for GWR with moving window kernel, b = 1200 km. AP: annual precipitation; MPDM: minimum precipitation of the driest month; WD: water deficit; MAT: mean annual temperature; MTCM: minimum temperature of the coldest month; PET: potential evapotranspiration; ΔAICC is the difference between the corrected Akaike information criterion values of two models; GWR: geographically weighted regression; OLS: ordinary least squares regression; *Best water model/best energy model. (DOC) [file pone.0027027.s004.doc]

**Table S3. Model selection for GWR with moving window kernel, *b* = 1200 km**.

| **Water** | | | **Energy** | | |  |  |
| --- | --- | --- | --- | --- | --- | --- | --- |
| **AP** | **MPDM** | **WD** | **MAT** | **MTCM** | **PET** | Δ**AIC_C_**  **to best GWR** | Δ**AIC_C_**  **to OLS** |
| × |  |  |  |  |  | 546 | 1138 |
|  | × |  |  |  |  | 437 | 2064 |
|  |  | × |  |  |  | 351 | 2460 |
| × | × |  |  |  |  | 258 | 1419 |
| × |  | × |  |  |  | 328 | 1342 |
|  | × | × |  |  |  | 135 | 2361 |
| × | × | × |  |  |  | 0* | 1648 |
|  |  |  | × |  |  | 898 | 1848 |
|  |  |  |  | × |  | 879 | 1385 |
|  |  |  |  |  | × | 813 | 1388 |
|  |  |  | × | × |  | 774 | 1058 |
|  |  |  | × |  | × | 766 | 1433 |
|  |  |  |  | × | × | 779 | 1284 |
|  |  |  | × | × | × | 641* | 1065 |

AP: annual precipitation; MPDM: minimum precipitation of the driest month; WD: water deficit; MAT: mean annual temperature; MTCM: minimum temperature of the coldest month; PET: potential evapotranspiration; ΔAIC_C_ is the difference between the corrected Akaike information criterion values of two models; GWR: geographically weighted regression; OLS: ordinary least squares regression; *Best water model/best energy model.
